# Post-thoracotomy pain relief with subpleural analgesia or thoracic epidural analgesia: randomized clinical trial: Alívio da dor pós-toracotomia com analgesia subpleural ou analgesia epidural torácica: ensaio clínico randomizado

**DOI:** 10.1590/1516-3180.2015.00462405

**Published:** 2015-11-13

**Authors:** Aysu Hayriye Tezcan, Özgür Karakurt, Mehmet Ali Eryazgan, Semih Başkan, Dilşen Hatice Örnek, Ramazan Baldemir, Bülent Koçer, Mustafa Baydar

**Affiliations:** I MD. Attending Physician, Anesthesiology and Reanimation Department, Ankara Numune Education and Research Hospital, Ankara, Turkey.; II MD. Attending Physician, Thoracic Surgery Department, Ankara Numune Education and Research Hospital, Ankara, Turkey.; III MD. Resident, Thoracic Surgery Department, Ankara Numune Education and Research Hospital, Ankara, Turkey.; IV MD. Associate Professor, Anesthesiology and Reanimation Department, Ankara Numune Education and Research Hospital, Ankara, Turkey.; V MD. Resident, Anesthesiology and Reanimation Department, Ankara Numune Education and Research Hospital, Ankara, Turkey.; VI MD. Associate Professor, Thoracic Surgery Department, Ankara Numune Education and Research Hospital, Ankara, Turkey.; VII MD. Attending Physician, Head of Department, Anesthesiology and Reanimation Department, Ankara Numune Education and Research Hospital, Ankara, Turkey.

**Keywords:** Pain, postoperative, Analgesia, epidural, Pleura, Thoracotomy, Analgesia, patient-controlled, Dor pós-operatória, Analgesia epidural, Pleura, Toracotomia, Analgesia controlada pelo paciente

## Abstract

**CONTEXT AND OBJECTIVE:**

Post-thoracotomy pain is a severe and intense pain caused by trauma to ribs, muscles and peripheral nerves. The current study aimed to compare subpleural analgesia (SPA) with thoracic epidural analgesia (TEA) in patients undergoing thoracotomy.

**DESIGN AND SETTING:**

Randomized study at Ankara Numune Education and Research Hospital, in Turkey.

**METHODS:**

Thirty patients presenting American Society of Anesthesiologists physical status I-III were scheduled for elective diagnostic thoracotomy. The patients were randomized to receive either patient-controlled SPA or patient-controlled TEA for post-thoracotomy pain control over a 24-hour period. The two groups received a mixture of 3 µg/ml fentanyl along with 0.05% bupivacaine solution through a patient-controlled analgesia pump. Rescue analgesia was administered intravenously, consisting of 100 mg tramadol in both groups. A visual analogue scale was used to assess pain at rest and during coughing over the course of 24 hours postoperatively.

**RESULTS:**

In the SPA group, all the patients required rescue analgesia, and five patients (33%) required rescue analgesia in the TEA group (P < 0.05). Patients who received subpleural analgesia exhibited higher visual analogue scores at rest and on coughing than patients who received thoracic epidural analgesia. None of the patients had any side-effects postoperatively, such as hypotension or respiratory depression.

**CONCLUSION:**

Thoracic epidural analgesia is superior to subpleural analgesia for relieving post-thoracotomy pain. We suggest that studies on effective drug dosages for providing subpleural analgesia are necessary.

## RESUMO

**CONTEXTO E OBJETIVO:**

Dor pós-toracotomia é severa e intensa, causada por trauma de nervos periféricos, músculos e costelas. O objetivo foi comparar analgesia subpleural (SPA) com analgesia epidural torácica (TEA) em pacientes submetidos à toracotomia.

**TIPO DE ESTUDO E LOCAL:**

Estudo randomizado no Hospital Educação e Pesquisa de Numune, em Ancara, Turquia.

**MÉTODOS:**

Trinta pacientes com o estado físico I-III da Sociedade Americana de Anestesiologistas foram agendados para toracotomia diagnóstica eletiva e escolhidos aleatoriamente para receber, num período de 24 horas, SPA ou TEA, ambas controladas pelo próprio paciente, para controle da dor pós-toracotomia. Os dois grupos receberam mistura de 3 ug/ml de fentanil com solução de 0,05% de bupivacaína por meio de bomba de analgesia controlada pelo paciente. Foi administrada analgesia de resgate por via intravenosa, com 100 mg de tramadol, nos dois grupos. No pós-operatório, a escala visual analógica foi utilizada para medir presença de dor durante a tosse ou em repouso, ao longo de 24 horas.

**RESULTADOS:**

No grupo SPA, todos os pacientes necessitaram de analgesia de resgate. Cinco pacientes (33%) necessitaram de analgesia de resgate no grupo TEA (P < 0,05). Os pacientes com SPA exibiram pontuações superiores na escala visual analógica, em repouso e ao tossir, em comparação aos que receberam TEA. Nenhum dos pacientes teve quaisquer efeitos secundários no pós-operatório, como hipotensão ou depressão respiratória.

**CONCLUSÃO:**

A analgesia peridural torácica é superior à analgesia subpleural no alívio da dor pós-toracotomia. Consideramos que estudos sobre a dosagem de drogas eficazes para proporcionar analgesia subpleural são necessários.

## INTRODUCTION

Post-thoracotomy pain is a severe and intense pain caused by trauma to ribs, muscles and peripheral nerves. Effective postoperative analgesia helps to reduce postoperative morbidity through early mobilization and rehabilitation and also reduces the development of chronic post-thoracotomy syndrome.^1^^,^^2^ Various analgesic techniques have been developed to treat postoperative thoracotomy pain.^1^^,^^2^^,^^3^^,^^4^^,^^5^^,^^6^ Thoracic epidural analgesia is the gold standard not only for pain relief after thoracotomy, but also because of its many beneficial effects, such as reduction of intraoperative opioid requirements, improvement of postoperative cardiopulmonary function and suppression of stress response.^1^^,^^2^^,^^3^^,^^4^^,^^5^^,^^6^ Thoracic epidural block is usually performed percutaneously, with considerable failure rates. Unfortunately, it is contraindicated in patients who are using anticoagulant or antiplatelet drugs.^7^ Intercostal nerve block, intrathecal administration of opioids and interpleural analgesia have also been developed as alternative regional techniques for post-thoracotomy pain management.^5^ Many of these techniques are claimed to provide good pain control, but studies to ascertain the ideal technique are still ongoing.^5^ There have not been enough studies on the subject of subpleural catheters for patient-controlled subpleural analgesia.

## OBJECTIVE

The current study aimed to compare subpleural analgesia (SPA) with thoracic epidural analgesia (TEA) in patient-controlled analgesia devices for patients undergoing thoracotomy.

## METHODS

This randomized clinical study included 30 patients with American Society of Anesthesiologists (ASA) physical status I-III, ranging in age from 20 to 70 years, for whom thoracotomy was planned. Approval for the study was granted by the institutional ethics board and written informed consent was obtained from all patients. Any patients with ASA status IV or greater, previous history of thoracotomy, use of chronic pain medication or contraindication against receiving local anesthetics or thoracic epidural block were excluded from the study. All the surgical procedures were performed by the same surgeons.

The patients were instructed how to use a patient-controlled analgesia (PCA) pump (Abbot Pain Management Provider, Abbott Laboratories, North Chicago, IL 60064, USA) and how to assess pain on a visual analogue scale (VAS), before their surgery. All the patients were pre-medicated with 1-2 mg of midazolam intravenously before surgery. The intraoperative monitoring included ECG, invasive arterial blood pressure, pulse oximetry, end-tidal carbon dioxide (EtCO_2_), end-tidal sevoflurane concentration and serial arterial blood gas (ABG) analysis.

**Figure 1.**
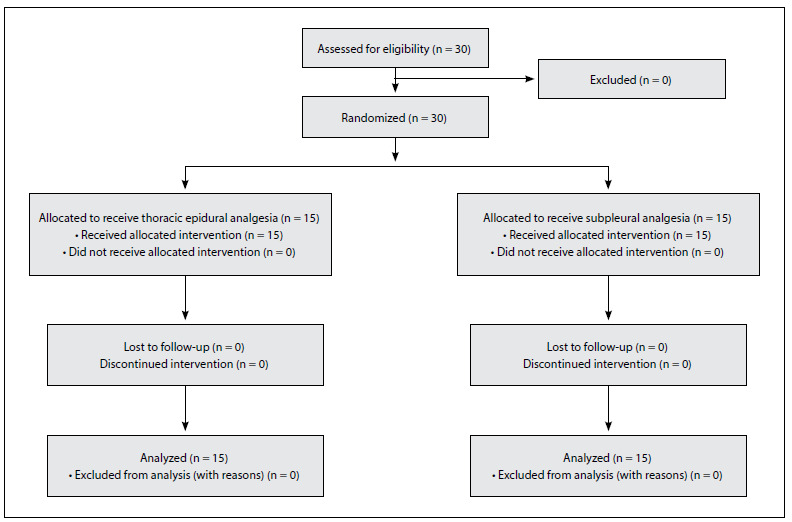
Patient inclusion and exclusion flowchart.

Patients were randomly assigned by means of the sealed envelope technique to either the thoracic epidural group (TEA group; n = 15) or the subpleural group (SPA group; n = 15). The patient inclusion and exclusion flowchart is described in Figure 1. Allocation was organized by a member of the medical staff who was not included in the study.

Anesthesia comprising fentanyl (2 µg/kg) and propofol (2-2.5 mg/kg) was induced and tracheal intubation was facilitated using 0.6 mg/kg of rocuronium. To maintain anesthesia, the patients received sevoflurane at 2% to 4% end-tidal concentration and 1 mcg/kg/h of remifentanil infusion, intravenously. All the patients were ventilated with a 50% oxygen and 50% air mixture. Muscle relaxation was obtained by means of a 0.1 mg/kg rocuronium bolus. After surgery, the neuromuscular blockade was reversed and the trachea was extubated in the operating room. All the patients were then transferred to the post-anesthesia care unit, where they were observed for 24 hours. All patients in this unit received O_2_ via a face mask at 0.4 FiO_2_ and were nursed in a 30° head-up position.

In the thoracic epidural anesthesia group (TEA group; n = 15), before induction of anesthesia, an epidural catheter was inserted in the thoracic region between T4 and T6 by an anesthesiologist, to a depth of 3-5 cm into the epidural space. The catheter placement was confirmed using 3 ml of 2% lidocaine with 1:200,000 adrenaline. Heparin and low molecular weight heparin therapies were stopped at least 6 or 12 hours, respectively, before the catheter insertion.

In the subpleural analgesia group (SPA group; n = 15), before the surgical wound was closed, the parietal pleura was removed bluntly from the posterior chest wall towards the vertebral body through three intercostal spaces above the thoracotomy incision. An 18-gauge epidural catheter was advanced into the space at the level of the neck of the ribs and laid on the endothoracic fascia under direct viewing. The catheter was secured with 4-0 prolene sutures to maintain its position during lung expansion and it extruded through the chest wall.

The TEA group received 10 ml of 0.125% bupivacaine and the SPA group received a loading dose of 20 ml of 0.25% bupivacaine via the catheter. In our department’s routine, PCA infusion is used postoperatively for all suitable patients, in order to achieve better analgesia outcomes. The two groups (SPA and TEA) received a mixture of 3 µg/ml of fentanyl with 0.05% bupivacaine solution through a PCA pump. The PCA pump was programmed to deliver at an infusion rate of 6 ml/h, and a bolus dose of 6 ml/h, with a locked-out interval of 15 minutes, and 60 ml within a 4-hour limit. In addition, all the patients received 75 mg of diclofenac sodium intramuscularly and 1 g of paracetamol intravenously every 12 hours. If required, 10 mg of metoclopramide was administered intravenously for nausea or vomiting. Rescue analgesia was administered consisting of 100 mg tramadol intravenously in both groups whenever the VAS score was > 4 at rest despite three consecutive PCA boluses. Absence of improvement in the VAS (VAS > 5) despite rescue analgesics was defined as analgesic failure. Pain intensity was measured at rest (VASr) and on coughing (VASc) using a visual analogue scale (0 = no pain; 10 = intolerable pain). The total tramadol doses were recorded over a 24-hour period postoperatively.

Preoperative baseline variables (heart rate, mean arterial blood pressure (MAP), PaO_2_, PaCO_2_ and respiratory rate) were recorded for each patient. These parameters together with analgesia and side effects (nausea/vomiting, pruritus, hypotension, respiratory depression and desaturation) were recorded in the post-anesthesia care unit at 0, 2, 8, 12 and 24 hours. Hypotension was defined as a drop in blood pressure of more than 25% of the baseline value. Respiratory depression was defined as respiratory rate of < 10/min. All the postoperative clinical outcome assessors and statistical analysis assessors were blinded. The member of the medical staff who monitored the PCA consumption, VAS scores and rescue analgesia requirements was blinded to the study.

### Statistics

To detect a difference from 80% to 30% in the incidence of analgesic failure, with a one-tailed significance level of 5% (α = 0.05) and β of 0.2 (power 80%), a sample size of 15 patients was required in each group.

Demographic variables (age, weight and height) and duration of surgery were compared using Student’s t test. Categorical variables were compared using the χ^2^ test. Pain scores, heart rate, mean arterial blood pressure (MAP), PaO_2_ and PaCO_2_ at different time intervals were compared using the Mann-Whitney U test. SPSS version 11.0 (SPSS Inc., Chicago, IL, USA) was used for all statistical analyses. P values < 0.05 were considered statistically significant.

## RESULTS

The demographic data from the two groups were similar with regard to age, height, weight, sex ratio and duration of surgery (Tables 1 and 2). The patients with patient-controlled subpleural analgesia (SPA group) had higher visual analogue scale scores (VAS) at rest (Table 3) and on coughing scores (Table 4) at all measurement times than the patients with patient-controlled thoracic epidural analgesia (TEA group).

**Table 1.**
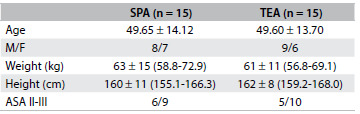
Demographic data and preoperative variables

Values are mean ± standard deviation (95% confidence interval) or number (P > 0.05). SPA = subpleural analgesia; TEA = thoracic epidural analgesia; M = male; F = female; ASA = American Society of Anesthesiologists.

**Table 2.**
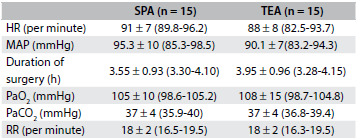
Postoperative variables

Values are mean ± standard deviation (95% confidence interval) or number. SPA = subpleural analgesia; TEA = thoracic epidural analgesia; MAP = mean arterial pressure, H = heart rate, RR = respiratory rate; P > 0.05.

**Table 3.**
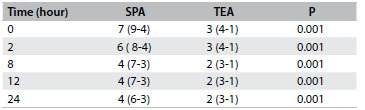
Visual analogue scale for pain at rest

Values are medians. SPA = subpleural analgesia; TEA = thoracic epidural analgesia.

**Table 4.**
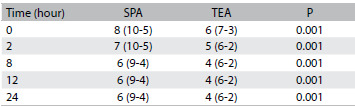
Visual analogue scale for pain during coughing

Values are medians. SPA = subpleural analgesia; TEA = thoracic epidural analgesia.

In the SPA group, all the patients required rescue analgesia using tramadol (100%). Five patients (33%) required rescue analgesia in the TEA group (P < 0.05). The mean dose of tramadol consumed as rescue analgesia postoperatively in the SPA group was 380 mg, compared with 120 mg in the TEA group (P = 0.002; Mann-Whitney U test).

The mean number of PCA boluses used was significantly lower in the TEA group: 7 in the TEA group versus 28 in the SPA group (P < 0.002; Mann-Whitney U test). The respiratory rate, heart rate, MAP, PaO_2_ and PaCO_2 values_ were comparable between the groups during the study period. None of the patients had hypotension or side effects. Oxygenation was satisfactory (PaO_2_ > 90 mmHg) in all the patients. None of the patients in either group showed respiratory depression (Table 2).

## DISCUSSION

The present study showed that SPA was not sufficiently effective for post-thoracotomy pain management. The patients using TEA with fentanyl and bupivacaine following thoracic surgery, even at low doses, had better analgesia both at rest and on coughing (Tables 3 and 4).

The mechanism of subpleural analgesia might be explained by the spread of local anesthetic to the posterior wall of the thorax, i.e. towards the vertebral column, and its diffusion to the paravertebral space, which contains the thoracic spinal nerves.^4^^,^^8^

Kanai et al. reported that subpleural analgesia provided successfully adequate pain control in two-thirds of their patients, through continuous infusion of 0.125% bupivacaine at 8 ml/h. However, in the current study, in the SPA group, all the patients required rescue analgesia. The failure to provide adequate post-thoracotomy pain relief could be attributed to dislodgement of the subpleural catheter or inadequate and limited diffusion of local anesthetic to the paravertebral space. The subpleural space is separated from the paravertebral space by the endothoracic or extrapleural fascia,^4^^,^^9^ and this barrier may prevent adequate diffusion of local anesthetic to the nerve endings.^4^^,^^10^ The deficiency of this study might be the low doses of local anesthetic usage, but there were not enough data about the ideal local anesthetic dosage for this kind of subpleural block, especially using bupivacaine, which has a long elimination time and cardiac side effects. Because of the unpredictable cardiac side effects, the concentration of bupivacaine used in this study was limited and fentanyl was added to the infusion solution to improve the analgesic quality.^11^^,^^12^^,^^13^

The most important advantages of patient-controlled epidural analgesia were the reduction of prolonged ventilation, reduction of re-intubation, improvements of pulmonary functions and early mobilization of the patient. The disadvantages of this technique were hypotension, urinary retention, pruritus and possible technical failure.^2^ Local anesthetic plus an opioid combination in PCA is believed to provide synergistic analgesia, thus requiring smaller doses and fewer side effects.^5^ Epidurally administered opioids produce segmental analgesia and improve the quality and duration of the sensory block produced by local anesthetics,^14^^,^^15^ which may explain the better pain relief in the TEA group. In our clinical practice, we usually use an opioid and local anesthetic mixture for TEA solutions and, even at low concentrations, adequate analgesia levels are obtained. As mentioned in relation to this current study, in clinical practice low doses of bupivacaine with TEA are sufficiently efficient to deal with post-thoracotomy pain.

Kanazi et al.^4^ determined that the pain scores when coughing were higher than at rest in all their patients and at all times, whether using TEA or SPA, and that VAS scores when coughing were always lower in the TEA group than in the SPA group. In that study, VAS scores at rest in the presence of thoracic epidural analgesia ranged from 1 cm to 6 cm. Those findings are similar to the findings of the current study (Tables 3 and 4).

There was no difference between the two groups of the current study in relation to the incidence of hypotension. However, Kanazi et al.^4^ reported that the incidence of hypotension was higher with thoracic epidural analgesia than with subpleural analgesia. This difference might be attributable to low concentrations of local anesthetic.

The strong point of the present study was that it showed that minimal bupivacaine doses were needed for effective thoracic epidural analgesia. The most important limitation of the study was the small sample size.

There are no published data identifying equipotent doses of bupivacaine for use in thoracic epidural and subpleural analgesia. Previous studies have suggested that local anesthetic doses for TEA should be half those of subpleural analgesia.^4^^,^^5^ In the current study, the starting bolus doses were given at a ratio of 1:2, but the PCA doses were lower than the doses used in the study by Kanazi et al. For supplemental therapy, paracetamol and diclofenac sodium were used in the current study. These bupivacaine doses in the TEA group were sufficient for analgesia, and reduced the rate of complications. However, they were not sufficient in the SPA group.

## CONCLUSION

In conclusion, TEA is better than SPA for providing post-thoracotomy pain relief. In order to avoid cardiac side effects of bupivacaine; the doses of the drug in the SPA group were limited and set as twice those of the TEA group. The local anesthetics and opioid doses in the TEA group in this study were safe and effective, but were insufficient in the SPA group even with parenteral supportive analgesic therapy. Subpleural analgesia with this dose regimen is not recommended for clinical practice. Further studies to determine local anesthetic doses and concentrations for this kind of subpleural analgesia are needed in order to achieve better analgesia for thoracic surgery.
